# Nucleocapsid Protein (N) of *Peste des petits ruminants* Virus (PPRV) Interacts with Cellular Phosphatidylinositol-3-Kinase (PI3K) Complex-I and Induces Autophagy

**DOI:** 10.3390/v15091805

**Published:** 2023-08-24

**Authors:** Yash Chaudhary, Juhi Jain, Sharad Kumar Gaur, Prabhakar Tembhurne, Shanmugam Chandrasekar, Muthuchelvan Dhanavelu, Sharvan Sehrawat, Rajeev Kaul

**Affiliations:** 1 Department of Microbiology, University of Delhi, South Campus, New Delhi 110021, India; yashchaudhary0210@gmail.com (Y.C.); jjain3101@gmail.com (J.J.); sharadgaur992@gmail.com (S.K.G.); 2 Department of Microbiology, Nagpur Veterinary College, Nagpur 440006, India; prabhakar.tembhurne@gmail.com; 3 Division of Virology, Indian Veterinary Research Institute, Mukteshwar, Nainital 263138, India; schand_vet@yahoo.co.in (S.C.); drchelva@gmail.com (M.D.); 4 Department of Biological Sciences, Indian Institute of Science Education and Research Mohali, Mohali 140306, India; sharvan@iisermohali.ac.in

**Keywords:** *Peste des petits ruminants* virus (PPRV), PPRV-N, PI3K complex-I, Autophagy, BECN1

## Abstract

Autophagy is an essential and highly conserved catabolic process in cells, which is important in the battle against intracellular pathogens. Viruses have evolved several ways to alter the host defense mechanisms. PPRV infection is known to modulate the components of a host cell’s defense system, resulting in enhanced autophagy. In this study, we demonstrate that the N protein of PPRV interacts with the core components of the class III phosphatidylinositol-3-kinase (PI3K) complex-I and results in the induction of autophagy in the host cell over, thereby expressing this viral protein. Our data shows the interaction between PPRV-N protein and different core components of the autophagy pathway, i.e., VPS34, VPS15, BECN1 and ATG14L. The PPRV-N protein can specifically interact with VPS34 of the PI3K complex-I and colocalize with the proteins of PI3K complex-I in the same sub-cellular compartment, that is, in the cytoplasm. These interactions do not affect the intracellular localization of the different host proteins. The autophagy-related genes were transcriptionally modulated in PPRV-N-expressing cells. The expression of LC3B and SQSTM1/p62 was also modulated in PPRV-N-expressing cells, indicating the induction of autophagic activity. The formation of typical autophagosomes with double membranes was visualized by transmission electron microscopy in PPRV-N-expressing cells. Taken together, our findings provide evidence for the critical role of the N protein of the PPR virus in the induction of autophagy, which is likely to be mediated by PI3K complex-I of the host.

## 1. Introduction

*Peste des petits ruminants* virus (PPRV) causes severe and highly contagious disease in both domestic and wild small ruminants, such as sheep and goats. PPRV belongs to the family *Paramyxoviridae* of the genus *Morbillivirus*, and its genome consists of negative-sense single-stranded RNA. The genome encodes six structural proteins, viz. the nucleocapsid protein (N), phosphoprotein (P), fusion protein (F), matrix protein (M), hemagglutinin-neuraminidase proteins (HN), and two non-structural proteins (C and V). In general, the viral proteins have been shown to be involved in the regulation of multiple cellular signaling pathways and work in sync to carry out functions that are critical for the propagation of the viruses in the host [1]. A recent study has shown that Canine distemper virus (CDV) N protein induced complete autophagy and facilitated viral replication [2]. Other studies have shown that the Heat shock protein 70K (HSP70K) interacts with the nucleoproteins (N) of CDV and measles morbillivirus [3,4].

In higher organisms, cell death can occur as a result of distinct processes, such as autophagy, apoptosis, and necrosis [5]. Autophagy is defined as an evolutionarily conserved intracellular process leading to the development of the double-membrane structures called autophagosomes. The process is not only involved in the degradation and recycling of misfolded or damaged organelles in the lysosomes [6,7], but also provides nutrients to cells under stress [8,9]. One of the primary targets of RNA viruses is the proteome associated with the process of autophagy, which may suggest the significance of autophagy regulation throughout the progression of viral infection [10]. Cellular protein phosphatidylinositiol-3-kinase complex-I is generated by phosphatidylinositol-3-phosphate (PI3P), which determines the sites for phagophore formation upon autophagy induction [11,12,13]. The PI3-kinase can be classified into two complexes: complex-I comprises vacuolar protein sorting 34 (VPS34), vacuolar protein sorting 15 (VPS15), Beclin-1 (BECN1), and ATG14L, while complex-II consists of VPS34, VPS15, BECN1, and ultraviolet (UV) irradiation-resistance-associated gene (UVRAG). Autophagy is modulated at different levels by various upstream signaling pathways regulated by VPS34 kinase [14]. The recruitment of PI3P-binding proteins occurs by PI3K kinase activity, subsequently leading to the conjugation of ATG8 family proteins, such as LC3, to phosphatidylethanolamine in phagophore membranes [15,16]. The autophagic flux or load can be correlated to the levels of lipid-conjugated microtubule-associated proteins 1A/1B light chain 3 or LC3 levels (LC3-II), with the level of LC3-II depicting the rates of LC3-II/autophagosome formation [17]. LC3 protein localizes in autophagic structures in the cells undergoing autophagy, and it is a mammalian ortholog of yeast ATG8 gene. There are three forms of LC3, i.e., LC3A, LC3B, and LC3C, among which LC3B is the most widely used marker for autophagic processes. LC3B is further processed by different ATG-family proteins into two forms: LC3-I and LC3-II. The conversion of LC3-I to LC3-II is also an important hallmark of autophagy [18]. Another important and widely used marker to study autophagic flux is the p62 protein, which is also called sequestosome 1 (SQSTM1). It binds to LC3 via a small LC3 interaction region (LIR). Its levels increase when autophagy in inhibited and decrease upon autophagy induction.

A stoichiometric subunit of PI3KC3 called Beclin-1-associated autophagy-related key regulator, or Barkor/ATG14(L), has been found to localize in autophagosomes and direct PI3KC3 to autophagosome. Several studies have described human ATG14 or Barkor protein as a potential functional mammalian ortholog of yeast ATG14. It has been shown to interact with Beclin-1 and the VPS34 class III PI3K complex to initiate autophagic vesicle nucleation [19,20,21,22]. Several reports have shown that many viruses modulate autophagy pathways of host cells in order to communicate with neighboring cells. For instance, the secretion, distribution, and transmission of infectious and non-infectious viral particles occur via the exosomal or secretory autophagy pathway [17]. Viruses modulate the autophagy pathway, resulting either in autophagy induction or autophagy inhibition upon infection. The outcome depends on the host cell, virus type, and cellular/environmental conditions [23]. Viruses code for proteins that can inhibit autophagy progression when autophagy is induced in infected cells as an anti-viral defense mechanism. In contrast, many viruses also have the ability to activate autophagy in host cells, allowing them to multiply and infect other cells [24,25]. Several reports have suggested that the autophagy pathway plays a crucial role in PPR virus replication in host cells, and thus, it might help in improving the efficiency of the production of live attenuated PPRV vaccines in a cell culture system by specifically targeting the autophagy pathway [26]. Autophagy induction by PPRV has been shown to result in the inter-cellular transmission of virus particles via the exosomal pathway. The study showed that TSG101 plays an important role in gathering infectious PPRV RNA genomes into exosomes, thereby aiding in release of virus particles [27]. The NECTIN 4 pathogen receptor has been revealed in another investigation to promote autophagy and PPRV viral replication. It has been shown that the expression levels of IRGM and HSPA1A were significantly increased following PPRV replication. However, the knockdown of these genes resulted in the impairment of a second wave of autophagy induced by PPRV [28]. Another study showed that the nucleocapsid protein of PPRV can inhibit interferon production mediated by interaction with IRF3, thereby blocking its activation [29]. Thus, the present study was undertaken with the aim to describe the role of *Peste des petits ruminants*-virus-coded N protein with phosphatidylinositol-3-kinase complex-I in the induction of autophagy.

## 2. Materials and Methods

### 2.1. Cell Lines and Cell Culture

Human embryonic kidney (HEK-293 and HEK-293T) and African green monkey (Vero) cells were used. HEK-293, HEK-293T, and Vero cells were maintained in Dulbecco’s minimal essential medium (DMEM; GIBCO, Thermo Fisher Scientific, Waltham, MA, USA; Cat. No. 12430-047) supplemented with 10% fetal bovine serum (FBS; GIBCO, Thermo Fisher Scientific, Waltham, MA, USA; Cat. No. 10270-106) 100 µg/mL penicillin, 100 µg/mL streptomycin antibiotics (Pan Biotech, Aidenbach, Germany; Cat. No. P06-07300), and 1 µg/mL Amphotericin B (HiMedia, Maharashtra, India; Cat. No. A011) in a tissue culture flask with 75 square centimeter growth surface. All the cells were incubated and propagated under standard conditions (37 °C, 5% CO_2,_ and 95% humidity).

### 2.2. Plasmids and DNA Transfections

The plasmid used to encode the Nucleocapsid (N) gene of PPRV Sungri/96 strain was a kind gift from the Indian Veterinary Research Institute (IVRI), Mukteshwar, Uttarakhand, India. The PPRV-N gene was amplified and sub-cloned into pCDNA-3X-flag vector, downstream of the CMV promoter. The pCR3.1-Beclin-1 (BECN1), pCAG-VPS34, pCAG-NT-VPS15, and pCAG-OSF 2X Strep/FL-ATG14L plasmid constructs were kind gifts from Prof. Beth Levine and Prof. Lyndaa Bennett, University of Texas South Western (UTSW) Medical Center, Texas, USA. Beclin-1 and ATG14L were amplified using specific primers and sub-cloned into eukaryotic HA-pCDNA expression vector. The primers used in the study have been listed in the Supplementary Table S1. For the expression analysis and further experiments, the cell lines were transfected using 1 mg/mL polyethylenimine (PEI, Polyscience, Inc. Warrington, PA, USA; Cat. No. 23966). For transfections, 2–3 million healthy cells were cultured for 24 h in 100 mm dishes. The plasmids to be transfected were diluted in 1 mL of 150 mM NaCl and 80 µL of PEI solution. The mixture was incubated at room temperature for 15–20 min before adding in the culture dishes. The medium was replaced with fresh medium after 3–4 h of transfection.

### 2.3. Immunoblotting Analysis

Whole cell lysates were prepared by harvesting the transfected cells and extracting the protein using RIPA lysis buffer (100 mM Tris-Cl, pH 7.5, 1 mM EDTA, 10% glycerol, 1% NP-40, and 150 mM NaCl) containing protease inhibitors (1 µg/mL pepstatin A (Cat. No. P5318), 1 µg/mL leupeptin, 1 µg/mL aprotinin (Cat. No. A1153), and 100 µM PMSF (Cat. No. P-7626) (Sigma Aldrich, St. Louis, MO, USA). Then, cell lysates were obtained by centrifugation at 14,000× *g* for 15 min at 4 °C. The protein concentration was estimated using Bradford’s reagent (Biorad; Cat No. 5000006), and approximately 80–100 µg of protein was separated on 12% SDS-PAGE gels. The protein bands were transferred onto polyvinylidene fluoride (PVDF) (mdi; Cat. No. SVFX8302XXXX101) membrane at 100 volts for 45–50 min, followed by blocking with 0.5% fat-free milk for 1 h at room temperature. The membrane was then washed with 1X TBST buffer thrice and incubated overnight with primary antibody (1:1000 dilution) at 4 °C. The next day, the membrane was thoroughly washed with 1X TBST thrice for 5 min each and incubated with fluorescently tagged Alexa-fluor goat anti-mice or goat anti rabbit secondary antibodies (1:10,000 dilution) for 1–2 h at room temperature. The fluorescence signal was measured using a Typhoon scanner (GE, Pittsburg, PA, USA) or FLA scanner (Fuji, Minato, Japan).

### 2.4. Antibodies and Reagents

As PPRV-N was sub-cloned into pCDNA-3X-flag-tagged eukaryotic expression vector, anti-flag monoclonal antibody was used in this study. The following primary antibodies were used in this study: anti-FLAG (Sigma-Aldrich, St. Louis, MO, USA; Cat. No. F3165), anti-HA (Cell Signaling Technology, Danvers, MA, USA; Cat. No. C29F4), anti-GAPDH (Cell Signaling Technology; Cat. No. 14C10), anti-GST (Thermo scientific, Waltham, MA, USA; Cat. No. 8-326), anti-PI3KC3 or anti-VPS34 (Cell Signaling Technology; Cat. No. 4263), anti-PI3KR4 or anti-VPS15 (Cell Signaling Technology; Cat. No. 14580), anti-ATG14L (Cell Signaling Technology; Cat. No. 96752), anti-LC3B (Affinity biosciences, Cincinnati, OH, USA; Cat. No. AF4650), and anti-SQSTM1/p62 (Affinity biosciences; Cat. No. AF5384). The following secondary antibodies were used in this study: Alexa Fluor goat anti-mice IgG 488 (Invitrogen, Carlsbad, CA, USA; Cat. No. A11001), Alexa Fluor goat anti-rabbit IgG 488 (Invitrogen; Cat. No. A11034), Alexa Fluor chicken anti-rabbit IgG 594 (Invitrogen; Cat. No. A21201). The following chemicals and reagents were used in this study: Nonidet P-40 (HiMedia, Maharashtra, India; Cat. No. RM2352), paraformaldehyde (Sigma-Aldrich; Cat. No. F8775), glutaraldehyde (Sigma-Aldrich; Cat. No. G6257), poly-L-lysine (Sigma-Aldrich; Cat. No. P4707), rapamycin (MedChem Express, Monmouth Junction, NJ, USA; Cat. No. HY-10219), chloroquine (Sigma-Aldrich; Cat. No. C6628), wortmannin (MedChem Express; Cat. No. HY-10197), and lysotracker red DND99 (Invitrogen; Cat. No. 7528).

### 2.5. GST-Tagged Protein Preparation

Only the PPRV-N-pGEX4T3 and pGEX-4T3 vector were transformed into *Escherichia coli* BL-21-competent cells. Then, they were plated on LB agar plates with ampicillin antibiotic and incubated at 37 °C overnight. A primary culture was propagated from a single colony in approximately 3 mL of Luria-Bertani (LB) broth medium supplemented with ampicillin antibiotic and incubated at 37 °C overnight at 200 rpm in a shaker incubator. The next day, 1% of the primary culture was added to 250 mL of LB medium with ampicillin and incubated at 37 °C with shaking at 200 rpm. The bacterial culture was incubated until the OD_600_ value reached 0.7, induced with 1 mM of final concentration of isopropyl β-D-1-thiogalactopyranoside (IPTG) (AMRESCO, Cat. No. 0487), and incubated further for 4 h at 37 °C with shaking at 200 rpm for protein over-production. After that, the bacterial cells were pelleted by centrifugation at 3000× *g* for 10 min at 4 °C, and the supernatant was discarded. The bacterial pellet was then washed twice with 10 mL of ice-cold STE buffer (100 mM NaCl, 10 mM Tris pH 7.5, and 1 mM EDTA) and centrifuged at 3000× *g* for 10 min at 4 °C. Next, the pellet was resuspended in 1.5 mL of ice-cold NETN buffer (100 mM NaCl, 20 mM Tris pH 8.0, 1 mM EDTA, and 0.5% NP-40) supplemented with protease inhibitors and incubated on ice for 15 min. Then, 75 µL of 1 M Dithiothreitol (DTT; Sigma-Aldrich; Cat. No. D9778) and 900 µL of 10% N-Lauryl sarcosine sodium salt (sarkosyl; Sigma-Aldrich; Cat. No. 137-16-6) prepared in STE buffer was added to the cell suspension, and the cells were lysed by 2 sonication cycles for 4 min each (10 secs ON/OFF pulse). The cell debris was pelleted by centrifugation at 10,000× *g* for 10 min at 4 °C after sonication. The supernatant was collected in a fresh tube, and 10% Triton X-100 prepared in STE and glutathione sepharose beads (GE Healthcare, Chicago, IL, USA; Cat. No. 17-0756-01) were added. The samples were then nutated at 4 °C overnight. The following day, the beads were centrifuged at 3000× *g* for 3 min at 4 °C. The beads were washed thrice with NETN buffer supplemented with protease inhibitors and resuspended in 100 μL of the same buffer. To check the expression of GST-tagged proteins, 10 μL of the beads were added to 10 μL of 4X SDS loading dye, and the sample was boiled for 5 min. The samples were then resolved by SDS polyacrylamide gel electrophoresis (SDS-PAGE) for the confirmation of protein expression. Only the concentration of pGEX-4T3 vector and GST-tagged PPRV-N protein was estimated according to the BSA protein standards.

### 2.6. GST Pulldown Assay

HEK-293T cells were used for the over-expression of PPRV-N or BECN1, or VPS34 or VPS15 or ATG14L. Lysates were prepared by transfecting approx. 10 × 10^6^ HEK-293T cells with PPRV-N or BECN1, or VPS34 or VPS15 or ATG14L expression plasmids and cultured for 48 h. The cells were then harvested, and protein lysate was prepared. Approx. 10% of the protein lysate was taken out from the total lysate, to be used as an input control. To the lysate, GST-tagged protein beads were added, and it was pre-cleared for 2 h at 4 °C in binding buffer supplemented with protease inhibitors. The lysate was then incubated with GST-tagged fusion protein beads. The following day, the beads were washed with the binding buffer (0.5% NP40, 20 mM Tris, 1 mM EDTA, 100 mM NaCl, 80% glycerol, and 5 mM DTT) supplemented with protease inhibitors (1 μg/mL pepstatin, 1 μg/mL aprotinin, 1 μg/mL leupeptin, and 100 μM PMSF) to remove any unbound proteins. The beads were then mixed with 4X SDS dye to extract and denature the bound proteins. The samples were loaded on to a 12% SDS-PAGE gel to resolve the protein bands, followed by Western blotting and probing using a tag-specific primary antibody.

### 2.7. Co-Immunoprecipitation (co-IP) Assay

HEK-293T cells were co-transfected with PPRV-N and BECN1 or VPS34, or VPS15, or ATG14L expression plasmids for 48 h. The transfected cells were harvested, and lysates were prepared using RIPA lysis buffer supplemented with protease inhibitors. From the total lysate, 5% of the lysate was taken out to be used as the input control. For each sample, 450 µL of lysate was incubated with 8 µg of primary antibody on nutation at 4 °C overnight. The next day, 50 µL of protein A (Thermo Scientific; Cat. No. 10-1141) and G beads (Thermo Scientific; Cat. No. 10-1242) were added in a ratio of 3:1, and the samples were kept again on nutation at 4 °C for 4 h. The beads were then centrifuged at 3000× *g* and washed thrice with RIPA (IP) buffer supplemented with protease inhibitors. The beads were then finally resuspended in 50 µL of IP buffer, and samples were prepared for SDS-PAGE by adding SDS loading dye. The samples were resolved on SDS-PAGE followed by immunoblotting.

### 2.8. Confocal Immunofluorescence Microscopy

Coverslips were treated with poly-L-lysine to be used in the experiment. Each treated coverslip was placed into each well of a 6-well plate. The HEK-293T cells were seeded in the 6-well plates with coverslips. After the cells were adhered to the coverslips, they were co-transfected with the expression plasmids of PPRV-N and BECN1 or VPS34, or VPS15 or ATG14L and incubated for 24 h. The cells were then washed gently with 1X PBS buffer three times after removing the medium. To fix the cells, 1 mL of methanol/acetone (1:1) was added to each well and incubated at −20 °C for 15 min. The cells were then air-dried and blocked with 1% fish skin gelatin (AMRESCO, Solon, OH, USA; Cat. No. M319) prepared in PBS. The coverslips were washed three times with 1X PBS buffer and incubated with specific primary antibodies at 4 °C overnight in a moist chamber. The next day, the coverslips were again washed with 1X PBS buffer three times and incubated with fluorescently labelled secondary antibodies for 2 h at room temperature in dark. Finally, the coverslips were washed again three times, and the cells were treated with DAPI (4′, 6-diamino-2-phenylindole; Sigma-Aldrich; Cat. No. D9542) for 30 s. The unbound DAPI was removed by washing three times with 1X PBS buffer. The coverslips were mounted onto the clean glass slides using fluoromount and visualized under a confocal microscope (Leica TCS SP8 system).

### 2.9. Quantitative Real-Time PCR (qPCR)

The total RNAs were extracted from the HEK-293 cells using an RNA isolation kit (Invitrogen, Carlsbad, CA, USA) as per the manufacturer’s protocol. The cDNAs were synthesized using a SuperScript First-Strand Reverse Transcription Kit (Agilent technologies, CA, USA) as per the manufacturer’s protocol. The primer pairs listed in the Supplementary Table S2 were used in RT-PCR for the amplification of TFEB, E2F1, BECN1, FOXO, GATA4, and ZKSCAN genes. Normalization was performed using GAPDH as an internal control. The experiments were performed independently in triplicates, and all experiments were carried out with at least three repeats.

### 2.10. Transmission Electron Microscopy

The visualization and evaluation of autophagosomes by transmission electron microscopy was performed at Sophisticated Analytical Instrumentation Facility (SAIF), AIIMS, New Delhi, India. In brief, the HEK-293T cells were mock-transfected or transfected with PPRV-N expression plasmid for 48 h. Rapamycin (200 nM)-treated HEK-293T cells were used as positive control. The cells were then harvested and centrifuged at 1000× *g* for 3 min. The cell pellet was washed with 0.1 M phosphate buffer pH 7.4. The cells were then fixed with 2% paraformaldehyde and 2.5% glutaraldehyde in 0.1 M phosphate buffer at 4 °C for 4–6 h. Next, the cells were washed and postfixed in 1% osmium tetroxide (O_S_O_4_) for 1 h at 4 °C. The samples were dehydrated in acetone, infiltrated and embedded in araldite CY212 (TAAB, Berkshire, UK). Ultrathin sections (1 µm) were cut using an ultramicrotome (Leica Ultracut UC7, Vienna, Austria) and mounted and adhered onto clean glass slides. Later, they were stained with aqueous toluidine blue. The sections were observed under the light microscope to check the quality of the tissue fixation. For electron microscope examination, thin sections of grey-silver color interference (70–80 nm) were cut and mounted onto 300 mesh-copper grids. The sections were stained with aqueous uranyl acetate and alkaline lead citrate for 10 min in each. They were washed gently with distilled water and observed under a Tecnai G2 20 high resolution transmission electron microscope (Fei Company, Eindhoven, The Netherlands) at an operating voltage of 200 kV.

### 2.11. Autophagic Flux Assay

For the autophagic flux assay, the HEK-293T cells were seeded in 6-well culture dishes, and cells were either transfected or mock transfected with PPRV-N expression plasmid, or treated with 200 nM of rapamycin for 48 h. The transfections or mock transfections with PPRV-N expression plasmid or rapamycin treatment was completed in two sets. One set of cells was used as control without chloroquine treatment, and the second set of cells was treated with chloroquine (20 µM) for 2 h. After that, cells were harvested and lysed using RIPA lysis buffer, and 100 µg of total protein lysate was resolved on SDS-PAGE, followed by immunoblotting using the appropriate primary antibodies to monitor autophagic flux. For the detection of the inhibition of autophagy, the HEK-293T cells were pretreated with 200 nM of wortmannin for 6 h, followed by transfection with PPRV-N expression plasmid and further incubation for 48 h at standard culture conditions. Later, the cells were harvested and lysed, and resolved on SDS-PAGE, followed by Western blotting with specific antibodies for autophagy.

### 2.12. Lysosome Staining

The HEK-293T cells were plated onto clean poly-L-lysine-treated coverslips placed in 6-well culture dishes. The cells were transfected or mock transfected with PPRV-N expression plasmid and culture for 48 h. The cells were gently washed with 1X PBS buffer and fixed with 3.2% of paraformaldehyde for 30 min at room temperature. For the lysosome staining, the cells were washed again with 1X PBS buffer and incubated with 100 nM lysotracker red DND-99 dye in DMEM medium for 30 min at 37 °C. Finally, the cells were counter-stained with DAPI after washing with 1X PBS buffer, mounted onto a clean glass slide, and finally visualized under a Carl Ziess Apotome 2 Axio Observer microscope (Göttingen, Germany) for lysosome detection. A total of three microscopic fields were observed for each sample, and the average red color intensity of lysotracker red dye was plotted on the graph. The experiments were performed independently in duplicates.

### 2.13. Imaging and Statistical Analysis

Confocal microscopy was performed using a Leica TCS SP8 confocal microscope, and Leica application suite advanced fluorescence software was used for the visualization of immune-stained cells and their quantification. The protein bands were quantified using ImageJ software (NIH). All the experiments were performed independently at least in duplicates or triplicates. Statistical analysis and the plotting of graph were performed using Microsoft Excel (MS office student 2016). The significance was determined by paired Student’s t-test. Values of *** p < 0.0005, ** p < 0.005, and * p < 0.05 were considered statistically significant.

## 3. Results

### 3.1. PPRV-N Protein Interacts with VPS34 of the Phosphatidylinositol-3-Kinase Complex-I In Vitro

**Figure 1:**
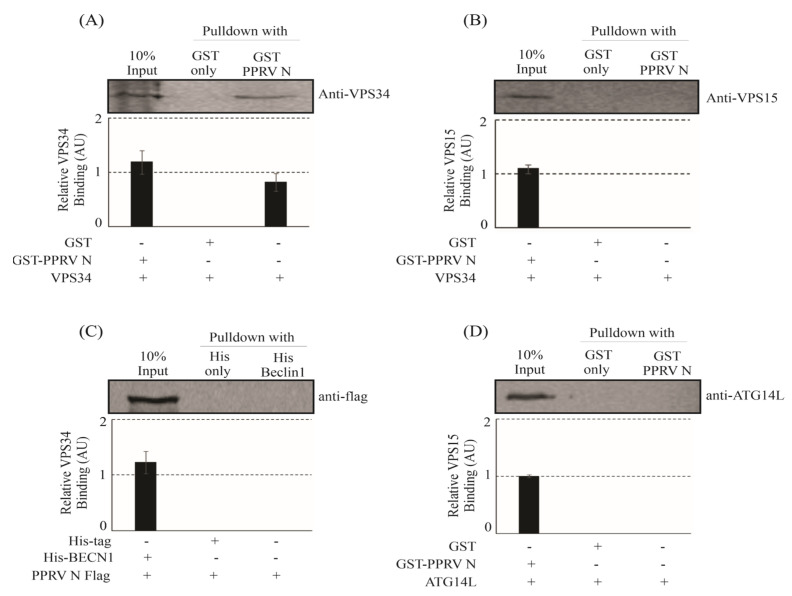
PPRVN interacts with VPS34 of the phosphatidylinositol−3−kinase complex−I in vitro. HEK−293T cells were used to transiently overexpress the core components of phosphatidylinositol−3−kinase complex−I, viz. VPS34, VPS15, BECN1, and ATG14L. GST pulldown assay was performed to test interaction between (**A**) GST−PPRV−N and VPS34−over−expressing cell lysate, (**B**) GST−PPRV−N and VPS15, (**C**) His−BECN1 and PPRV−N, and (**D**) GST−PPRV−N and ATG14L. The experiment was performed in triplicates, and the graph shows the error bars that depict the standard deviation from the triplicates.

Autophagy is a well-known mechanism in the cell designed to regulate diverse cellular functions, including growth, differentiation, response to nutrient deficiency and oxidative stress, cell death, and pathogen infection. Several studies have shown that autophagy can be modulated at different steps that involve different proteins or a complex of proteins. The PI3K complex-I is one such protein complex involved in autophagy that comprises PIK3C3 or VPS34, PIK3R4 or VPS15, Beclin-1 or BECN1, and ATG14L [30]. PPRV is known to play role in the induction of autophagy in infected cells. Since N protein is the most abundant protein constituting the virus, we therefore wanted to test if PPRV-N can interact with the components of PI3-kinase complex-I as this complex plays a crucial role in the initiation of autophagy. The GST pull down approach was used to test if the components of PI3K complex-I can interact with PPRV-N protein. In the GST pull down assay, the components of PI3K complex-I (i.e., VPS34, VPS15, Beclin-1, and ATG14L) were separately ectopically over-expressed in HEK-293T cells and tested for their interaction with GST-PPRV-N protein. Our results indicate that only VPS34 was able to bind efficiently to the bacterially expressed and purified GST-PPRV-N (Figure 1A). However, none of the remaining three components of the PI3K complex-I were able to bind to GST-PPRV-N-expressing sepharose beads (Figure 1B–D). Therefore, our data clearly shows that the PPRV-N protein can interact with VPS34, which is a part of the PI3-kinase complex-I.

### 3.2. PPRV-N Protein Interacts with Phosphatidylinositol-3-Kinase Complex I In-Vivo

**Figure 2:**
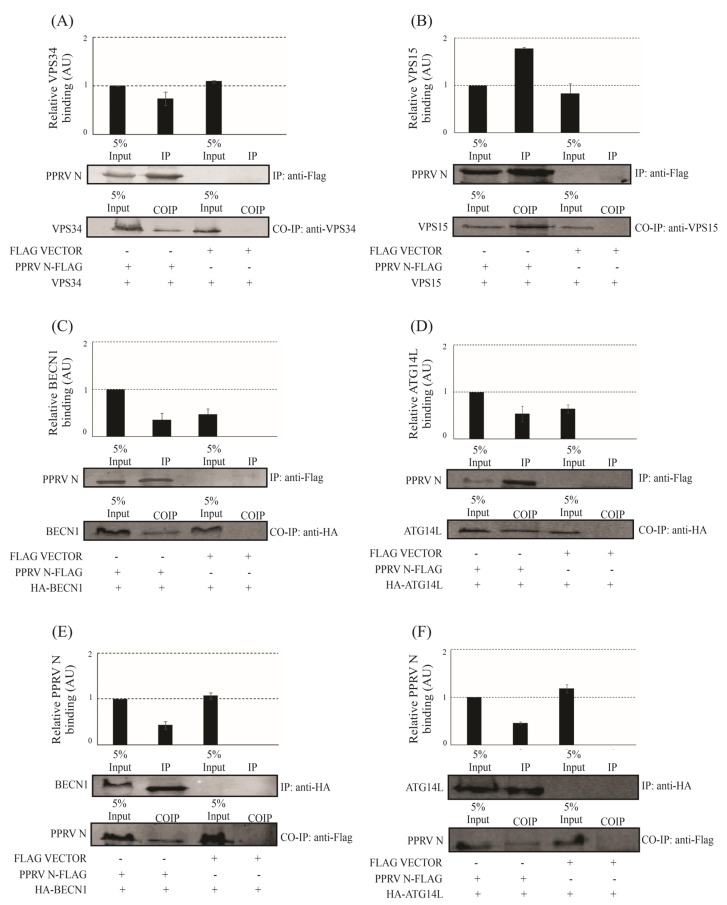
PPRV−N interacts with components of phosphatidylinsitol−3−kinase complex−I in vivo. PPRV−N, along with core components of phosphatiylinositol−3−kinase complex−I, viz. VPS34 or VPS15, or BECN1 or ATG14L, were ectopically over-expressed in HEK−293T cells, and an immunoprecipitation experiment was performed to test their interactions using tag-specific or protein-specific antibodies, as indicated in figure panels. (**A**) PPRV−N−flag interaction with VPS34, (**B**) PPRV−N−flag interaction with VPS15, (**C**) PPRV−N−flag interaction with BECN1, (**D**) PPRV−N−flag interaction with ATG14L, and (**E**,**F**) the reciprocal co-immunoprecipitation was performed with anti-HA antibody, where PPRV−N−flag was co-expressed with HA−BECN1 and HA−ATG14L, separately. The reciprocal CoIP was detected using anti-flag antibody. The experiment was performed in triplicates, and the graph shows the error bars that depict the standard deviation from the triplicates.

Next, we performed a co-immunoprecipitation assay to investigate whether or not PPRV-N can interact with the core components of PI3-kinase complex-I in vivo. VPS34 is the core component of PI3-kinase complex-I, and other components, including VPS15, Beclin-1, along with ATG14L, constitute PI3K complex-I, which plays an essential role in autophagosome biosynthesis [31]. To test these interactions in vivo, PPRV-N and the components of PI3K complex-I were individually co-overexpressed in HEK-293T cells, followed by a Co-IP assay using tag-specific antibodies or protein-specific antibodies against VPS34 or VPS15, or BECN1 or ATG14L. Our results indicate that all four components of PI3K complex-I co-immunoprecipitated with PPRV-N protein (Figure 2). The interactions of PPRV-N protein with VPS34 or VPS15 (Figure 2A,B), were stronger in comparison to its interaction with BECN1 or ATG14L (Figure 2C,D) as visible in the Western blots. A previous study, in which the crystal structure of PI3K complex-I was resolved, had shown that VPS34 and VPS15 proteins are more closely associated with each other than with other components in this complex [32]. Our data, showing a relatively stronger interaction of PPRV-N protein with VPS34 and VPS15, indicates that the interaction of PPRV-N with PI3K complex-I is likely to mainly involve the part of this complex that is made up of VPS34 and VPS15 protein subunits. In addition, the reciprocal CoIP assay to test the interaction between PPRV-N and BECN1 or ATG14L also confirmed the interaction between the viral and the host proteins (Figure 2E,F). Therefore, our data clearly indicate that PPRV-N protein interacts stably and efficiently with the core components of PI3-kinase complex-I.

### 3.3. PPRV-N Colocalizes with the Components of Phosphatidylinositol-3-Kinase Complex-I, inside Cell

**Figure 3:**
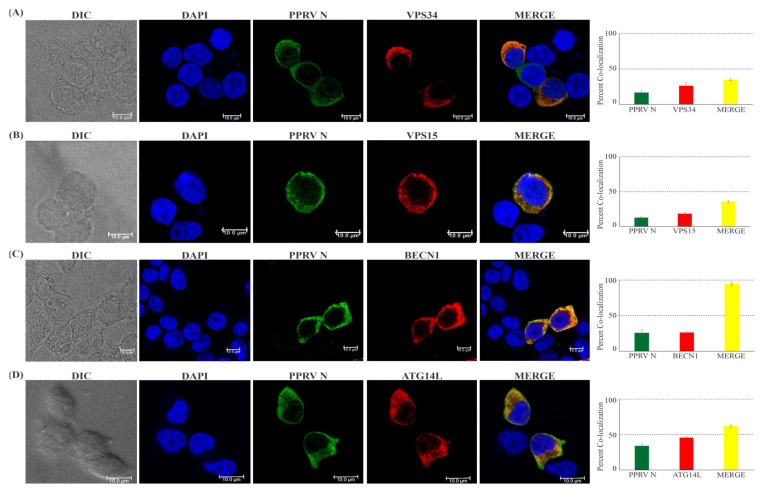
PPRV−N protein colocalizes with each component of phosphatidylinositol−3−kinase complex−I. The intracellular localization of ectopically expressed PPRV−N structural protein and phosphatidylinositol−3−kinase complex−I components were analyzed by confocal microscopy. HEK-293T cells were co-transfected to over-express PPRV−N with each component of PI3−kinase complex-I individually for 24 h. Cells were fixed and subjected to indirect immunofluorescence analysis using antibodies against Flag-tag (green), HA-tag (red), VPS34 (red), and VPS15 (red). The nuclei of the cells were counterstained with DAPI. PPRV−N can colocalize with (**A**) VPS34, (**B**) VSP15, (**C**) BECN1, and (**D**) ATG14L in the cytoplasm that has been depicted in yellow. However, no remarkable effect on the trans-localization of the host protein was seen (scale bar: 10 µm).

The interaction of viral protein with cellular host proteins may result in changes or a shift in their intracellular localization. In order to test whether PPRV-N and the components of PI3K complex-I colocalize in the same intracellular compartment, or if their interaction results in any significant changes in their sub-cellular localization, we performed an indirect immunofluorescence assay using confocal microscopy. Our data shows that PPRV-N protein colocalizes with core components of PI3-kinase complex-I in the cytoplasm (Figure 3). The colocalization of PPRV-N was ~35% with VPS34 (Figure 3A), ~35% with VPS15 (Figure 3B), ~90% with Beclin-1 (Figure 3C), and ~60% with ATG14L (Figure 3D). There were no evident changes or alterations in the intracellular trans-localization of either the viral or the host protein as a result of these interactions. Thus, our data clearly suggests that the PPRV-N, as well as these host proteins involved in autophagy-related pathways, were predominately colocalized in the cytoplasm in the same sub-cellular compartment without having any effect on the trans-localization of any protein from cytoplasm to nucleus.

### 3.4. PPRV-N Modulates the Transcription of Autophagy-Related Genes

**Figure 4:**
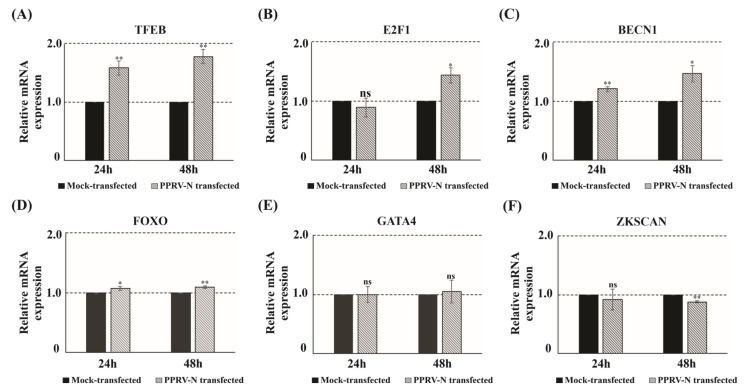
PPRV−N expression results in the up-regulation of transcript levels of genes involved in autophagy induction and the down-regulation of transcript levels of genes involved in autophagy repression. The effect of PPRV−N over-expression on the transcription of autophagy-related genes was tested by performing quantitative real time PCR. HEK−293 cells were transfected or mock transfected with PPRV−N expression plasmid and cultured for 24 or 48 h. The total RNA was isolated at given time points, followed by cDNA synthesis. The cDNAs were subjected to quantitative−RT PCR analysis to check the transcriptional level of autophagy-related genes. Data are expressed as the mean SEM of three independent experiments. ** p < 0.005, * p < 0.05; ns: non-significant.

Autophagy can be induced by a variety of stimuli, like mechanical stress, nutrient depletion or starvation, hypoxia, DNA damage, chemical compounds, and pathogen infection. It is associated with the transcriptional modulation of various autophagy-related genes (ATGs). Studies have shown that the induction of autophagy involves the upregulation of several transcriptional factors, such as transcription factor EB (TFEB), E2 transcription factor 1 (E2F1), Forkhead box O (FOXO), Beclin-1 (BECN1), and the down-regulation of GATA4 and ZKSCAN [33,34]. To investigate whether or not the expression of PPRV-N gene has any significant effect on the transcription of these autophagy-related genes, quantitative real time PCR was performed. Our data indicate that the genes known to induce autophagy were found to be up-regulated in the PPRV-N-expressing cells (Figure 4). The data shows a significant up-regulation in the transcript levels of TFEB (Figure 4A) at both 24 and 48 h, while E2F1 (Figure 4B) and BECN1 (Figure 4C) showed up-regulation only after 48 h. However, FOXO (Figure 4D) did not show any significant up-regulation either at 24 or at 48 h. TFEB transcript levels were up-regulated approximately 1.8 fold, whereas E2F1 and BECN1 levels were increased approximately 1.5 fold, at 48 h. In contrast, the genes responsible for the repression of autophagy, i.e., GATA4 (Figure 4E) and ZKSCAN (Figure 4F), were found to be either unaffected or showing a low level of down-regulation. GATA4 was found to remain unaffected in the presence of PPRV-N gene expression at both 24 and 48 h, while ZKSCAN showed a lower level of down-regulation (only 10%) at 48 h. Thus, our findings show that the expression of autophagy-related genes was affected in PPRV-N-expressing cells, thereby indicating the role of PPRV-N in the induction of autophagy.

### 3.5. PPRV-N Protein Expression Enhances the Autophagic Flux

Autophagy is associated with the activation of multiple autophagy-related genes. LC3B and SQSTM1/p62 proteins have been widely used to study the autophagic flux. We tested the endogenous levels of LC3-II and SQSTM1/p62 using specific antibodies in cells over-expressing PPRV-N protein. Rapamycin acts by inhibiting the mammalian target of rapamycin (mTOR) and is considered as a positive inducer of autophagy [35,36]. Therefore, we used rapamycin-treated cells as a positive control for the induction of autophagy.

**Figure 5:**
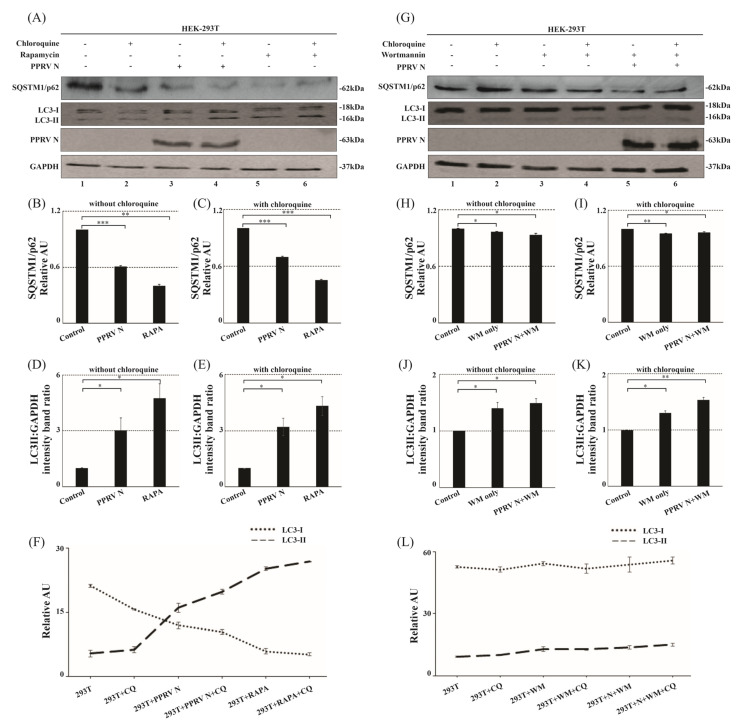
PPRV−N expression increases the conversion of LC3−I to LC3−II in HEK−293T cells and concurrently decreases SQSTM1/p62 levels, mediated by phosphatidylinositol−3−kinase complex−I. (**A**) HEK−293T cells were transfected with PPRV−N expression plasmid or treated with rapamycin for 48 h. The LC3−II levels in the cells treated with or not treated with chloroquine were compared between control cells and either PPRV−N−expressing cells or rapamycin-treated cells. Corresponding graphs depict the levels of SQSTM1/p62 (**B**,**C**) and LC3B−II (**D**,**E**). (**F**): The line graph showing the comparison between SQSTM1/p62 and LC3B−II, in the absence and presence of chloroquine. (**G**): HEK−293T cells were pretreated with wortmannin for 6 h and transfected with PPRV−N expression plasmid for 48 h. The LC3B conversion and SQSTM1/p62 levels were compared both in the presence and absence of chloroquine between mock transfected and PPRV−N−transfected cells. Corresponding graphs depict the levels of SQSTM1/p62 (**H**,**I**) and LC3B−II (**J**,**K**). (**L**): The line graph showing the comparison between SQSTM1/p62 and LC3B−II in the absence and presence of wortmannin. GAPDH was used as the internal loading control. Antibodies against SQSTM1/p62 and LC3B were used in immunoblotting analysis. The data represents the mean ± SD of three independent experiments. Paired Student’s *t*-test was used to determine the significance. *** p < 0.0005, ** p < 0.005, * p < 0.05; ns: non-significant.

Our data shows that in PPRV-N-over-expressing cells, there was a decrease in SQSTM1/p62 levels (Figure 5A). The relative amount of LC-3-II in comparison to LC3-I was significantly higher in PPRV-N-over-expressing cells compared to the control, indicating that PPRV-N expression was associated with the initiation of the autophagy process in the cells. Similar observations were made in the cells treated with rapamycin. The quantification of protein bands, depicted in one of the following graphs (Figure 5B), show the significantly reduced levels of SQSTM1/p62 both in the absence (Figure 5B; compare lanes 1, 3, and 5) and presence of chloroquine (Figure 5C; compare lanes 2, 4, and 6). In addition, the LC3-II levels were significantly up-regulated both in the absence (Figure 5D; compare lanes 1, 3, and 5) and the presence of chloroquine (Figure 5E; compare lanes 2, 4, and 6). The line graph comparing the levels of SQSTM1/p62 and LC3-II in PPRV-N-over-expressing cells with or without the treatment of chloroquine shows the increased autophagic flux in the PPRV-N-over-expressing cells (Figure 5F). The expression of the endogenous or intracellular LC3B levels in control cells were observed to be at the basal level, both in the presence and absence of chloroquine (Figure 5A, lanes 1 and 2). Our findings indicate that the SQSTM1/p62 levels were reduced, and LC3-II conversion was increased in both PPRV-N-expressing or rapamycin-treated cells. Thus, our data indicates that there was a significant increase in the autophagic flux in the cells over-expressing PPRV-N protein.

### 3.6. Inhibition of Phosphatidylinositol-3-Kinase Complex-I in PPRV-N Over-Expressing Cells Rescues the Cells from Autophagy

To further validate the role of PI3-kinase complex-I in PPRV-N-mediated autophagic flux, we pre-treated the PPRV-N-over-expressing cells with wortmannin. Wortmannin is a potent inhibitor that inhibits the activity of PI3-Kinase complex-I and halts the initiation of autophagosome biosynthesis in the autophagy pathway [37]. Our data shows that pre-treatment of PPRV-N-over-expressing cells with wortmannin allowed for the rescue of the cells from the induction of autophagy, as indicated by the increased expression of SQSTM1/p62 (Figure 5G; compare lane 5 with lane 6 in the top panel) and by the reduced conversion of LC3-I to LC3-II (Figure 5G; compare lane 5 with lane 6 in the second panel). The quantification of these proteins has been depicted in Figure 5H,I (for SQSTM1/p62 protein) and Figure 5J,K (for LC3-II protein). The line graph depicts the minimally affected levels of SQSTM1/p62 and reduced LC3-II conversion in PPRV-N-over-expressing cells upon treatment with wortmannin (Figure 5L). Hence, our data clearly shows a direct role of phosphatidylinositol-3-kinase complex-I in the induction of autophagy in PPRV-N-over-expressing cells.

### 3.7. PPRV-N Expression Induces Autophagy Associated Changes in Cells in a Dose-Dependent Manner

**Figure 6:**
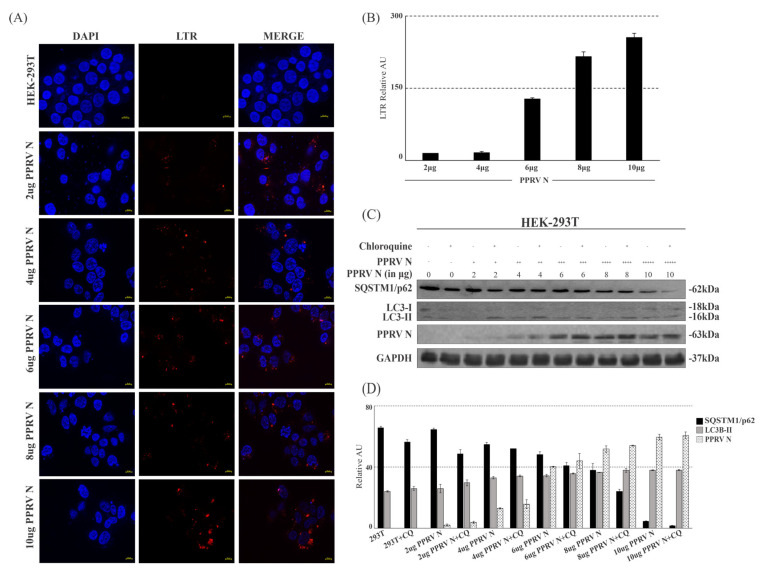
PPRV−N expression results in autophagy induction in a dose-dependent manner. The effect of increasing expression of PPRV−N protein was tested by immunofluorescence assay and Western blotting. (**A**) HEK−293T cells were transfected with increasing amounts of PPRV−N expression plasmid for 48 h, followed by an immunofluorescence analysis and the detection of lysosome formation using lysotracker red dye. (**B**) The corresponding graph depicts the quantification of red-color representing the lysosome formation. The lysotracker detection experiment was performed in duplicates. (**C**) Western blot analysis was performed upon transfection with PPRV−N expression plasmid in increasing concentrations in HEK−293T cells for 48 h. Autophagic flux was determined using antibodies against LC3B and SQSTM1/p62. (**D**) Corresponding graph showing the comparison between SQSTM1/p62 and LC3B−II levels in the presence of an increasing expression of PPRV−N protein. LTR: Lysotracker.

Lysosomes containing hydrolytic enzymes are membrane-bound organelles that play key roles in a number of cellular functions, including metabolism, plasma membrane repair, autophagy, cell signaling, and biomolecule degradation. They digest extracellular material through endocytosis and intracellular material through autophagy. Lysosome staining can be employed to detect autophagy by recognizing and tracking lysosomes with lysosome markers, which localize within lysosomes [38,39]. Lysotracker dyes (red or green) are most commonly and widely used to stain lysosome. Therefore, to visualize and study autophagy-associated lysosomal activity, we performed lysosome staining on cells over-expressing increasing amounts of PPRV-N protein. Our data shows the substantially increased accumulation of lysotracker stain in the cells expressing PPRV-N protein, whereas no accumulation of lysotracker dye was observed in the HEK-293T control cells (Figure 6A). The corresponding graph depicted that the abundance of lysosomes was increased upon the increase in PPRV-N protein expression in a dose-dependent manner (Figure 6B). We further detected the expression of endogenous or intracellular SQSTM1/p62 and LC3B in these cells to ascertain the fact that an increasing PPRV-N expression results in increased autophagy. Our results show a remarkable reduction in SQSTM1/p62 levels and increased LC3-II levels in cells over-expressing PPRV-N protein in a dose-dependent manner (Figure 6C,D). Taken together, our data indicates that the accumulation of lysotracker stain in lysosomes, or the lysotracker red signal and the formation of autophagosomes, were considerably increased with the increasing concentration of PPRV-N in a dose-dependent manner.

### 3.8. Visualization of Autophagosomes in Cells Expressing PPRV Nucleocapsid (N) Protein

**Figure 7:**
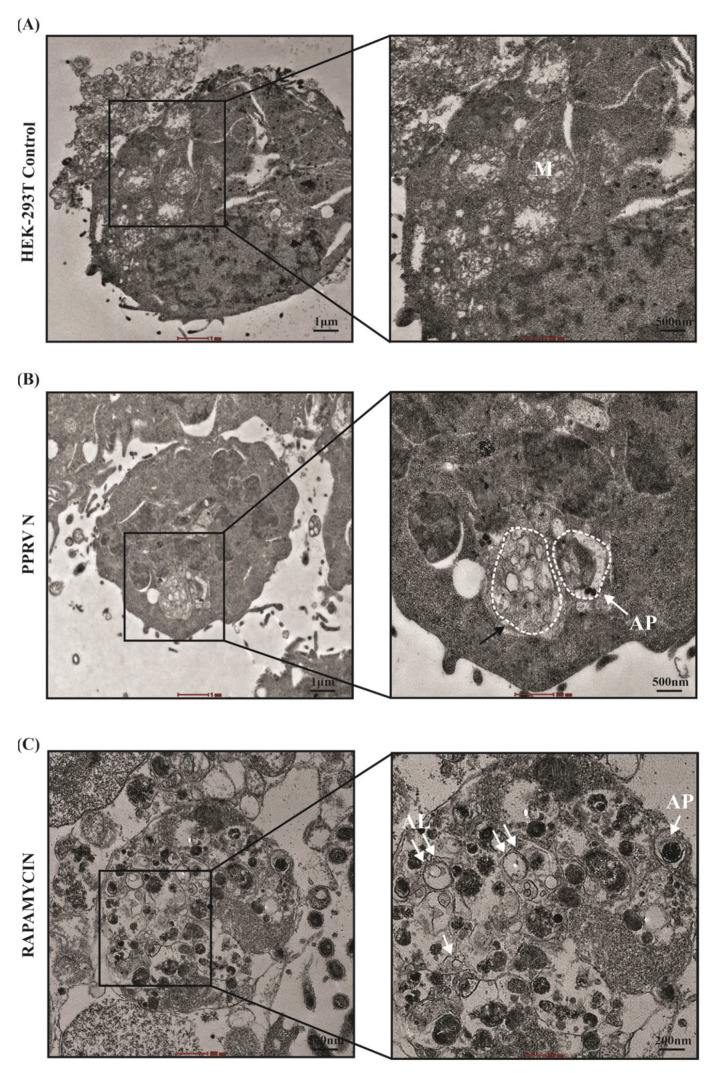
PPRV−N protein expression results in the formation of autophagosomes. HEK−293T cells were transfected with PPRV−N expression plasmid for 48 h. Cells treated with rapamycin (200 nM, 24 h) were used as positive control. After 48 h, the cells were processed and examined by TEM. (**A**) Untreated or mock transfected cells. (**B**) Cells expressing PPRV−N. (**C**) Cells treated with rapamycin. Boxed areas were further enlarged. Single white arrows indicate autophagosomes (AP), double arrows indicate autolysosomes (AL), and the black arrow indicates a large autophagosome with multiple organelles (partially degraded). M: Mitochondria.

Autophagy or autophagic structures can be best visualized with electron microscopy techniques. Transmission electron microscopy is one of the many methods used to confirm the formation of autophagic structures, such as autophagosomes and autolysosomes. Our analysis of TEM images show the formation of single- and double-membrane vesicles designated as autophagosomes, which were observed in PPRV-N-over-expressing cells. Giant or large autophagosome with multiple organelles (partially degraded) were also observed in the presence of PPRV-N (Figure 7B). These were similar to the large number of autophagic vesicles or autophagosomes which were observed in the rapamycin-treated positive control cells (Figure 7C). In contrast, notably lower numbers of vesicles were observed in the untreated or mock-transfected HEK-293T cells, which showed dense cytoplasm and mitochondria (Figure 7A). Therefore, our data from the transmission electron microscopy assay clearly indicates that the expression of PPRV-N in cells results in autophagosome biosynthesis.

## 4. Discussion

*Peste des petits ruminants* virus causes a highly contagious disease of great economic importance. The disease is considered to be a potential threat to small ruminants around the globe [40]. Upon infection of the intracellular pathogens, host cells can induce autophagy as an innate immune response to control the spread and transmission of the disease. Several studies have shown that autophagy can play an important role in both the protective function for host cell survival under stress, and also in the cellular defense mechanism against the pathogen [41,42]. VPS34 protein is considered to be the core subunit of the class III PI3K-complex-I, and its main function includes the activation of the autophagy pathway. VPS15 is believed to anchor VPS34 to the membrane through N-terminal myristylation and acts as a positive regulator of autophagy [43,44]. Beclin-1 is another regulatory subunit of this complex that plays an important role in binding to other modulators of autophagy. ATG14L is another regulatory protein present at the peripheral ER and forms puncta during autophagosome formation [22,45].

Our study has now shown that PPRV-coded structural protein N can interact with the components of phosphatidylinositol-3-kinase complex-I in vitro (Figure 1) as well as in vivo (Figure 2). Previous reports have shown that ATG14L/Barkor interacts with Beclin-1 and VPS34, and together, they play an important role in the initiation of the autophagy process [19,20,21,22]. Beclin-1 is known as an autophagy marker and is known to interact with various proteins of viruses. Earlier, studies have also shown that the Nef protein of HIV interacts directly with Beclin-1, thereby inhibiting the autophagy pathway, which was further confirmed by assessing the mRNA expression levels [46]. A study had shown that the NS2 protein of human respiratory syncytial virus induces autophagy through the modulation and stabilization of Beclin-1 protein by impairing ISGylation to overcome autophagy inhibition [47]. Studies had also shown that HCV non-structural protein NS4B interacts and forms a complex with Rab5 and VPS34 that leads to autophagy induction [48]. The HCV non-structural protein 5A interacts with ATG14L and ATG14L-Beclin1-VPS34-VPS15 complex, which is necessary for autophagosome formation, in a RACK1-dependent manner. The interaction is important for the formation of HCV double-membrane vesicle and viral replication [49]. Another study showed nuclear receptor binding factor 2 (Nrbf2) interacts with ATG14L-containing Beclin-1-VPS34 complex and plays an important role in autophagic regulation [50]. VPS34 acts as a catalytic subunit, which exists in a heterodimeric structure with the regulatory subunit, VPS15 [51]. Therefore, VPS34 is considered to be the core of the PI3K complex-I, and the interaction of PPRV-N with VPS34 signifies its probable contribution in the initiation of the autophagy pathway. Our study now shows the evidence for a stable interaction between PPRV-N and PI3kinase complex-I protein components, and that this interaction may be simply involved in the initiation of autophagosome biosynthesis. Thus, the interaction of PPRV-N with individual components of phosphatidylinositol-3-kinase complex-I specifies the critical role of PPRV-N in initiating the formation of autophagosomes and hence autophagy.

We also investigated the sub-cellular localization of these different components of phosphatidyl-3-kinase complex-I in cells expressing PPRV-N viral protein. Previous research has demonstrated that in RPE-1 cells, ectopically expressed mCherry-Nrbf2 and Atg14L-EGFP predominantly colocalized on punctate structures under both nutrient-rich and serum-starved conditions [50]. The Rab7 protein, which is essential for transport between early and late endosomes, colocalizes with p150/VPS15 on punctate perinuclear vesicles. In order to form a complex on late endosomes, it also colocalizes with hVPS34 [52]. Our findings showed that the PPRV-N protein and the host proteins involved in autophagy, largely colocalizes in the cytoplasm without having any remarkable effect on their intracellular localization. The presence of PPRV-N protein does not cause any change in the expression and localization of the host proteins. The fact that PPRV-N and PI3-kinase complex proteins exist stably inside the same sub-cellular compartment could be important for their stable interaction, which would be critical for the role of these interactions in the modulation of the autophagy pathway.

The process of autophagy is very much regulated and involves the modulation of the transcription of several transcriptional factors. The first observation related to the role of transcriptional regulation in autophagy induction was made in yeast by Kirisako group, where they reported that nitrogen starvation in yeast can trigger autophagy by up-regulating the Apg8p autophagy gene, which is also a homolog of mammalian LC3 [53]. In past, several studies have reported the up-regulation or down-regulation of several autophagy-related genes, which correlate with the induction of autophagy. Our study also showed that the transcription of several autophagy inducers, like TFEB, E2F1, BECN1, and FOXO, increased significantly in the presence of PPRV-N protein; while the transcription of autophagic repressors, like ZKSCAN and GATA, were either reduced or remained unaffected. Therefore, the real time data implies that PPRV-N ectopic expression can modulate the transcription of genes involved in the induction and repression of autophagy.

For monitoring the autophagic flux in the cultured cells, LC3 has been used widely as an autophagosome marker. In our study, there was a significant increase in the accumulation of LC3-II and a simultaneous reduction in the levels of SQSTM1/p62 in PPRV-N-expressing or rapamycin-treated cells, both in the presence and absence of chloroquine. These findings are correlate with the significant contribution of PPRV-N in triggering the formation of autophagosomes mediated by phsophotidylinositol-3-kinase complex-I, thereby leading to enhanced autophagic activity. In contrast, low basal levels of LC3-II and SQSTM1/p62 were detected in the mock transfected HEK-293T cells, indicating the basal level of autophagy. Our study further showed that the initiation of autophagy in PPRV-N-expressing cells was blocked after treatment with wortmannin, as indicated by reduced accumulation of LC3-II, resulting in reduced autophagic flux. On the other hand, there were no significant changes in the mock transfected or untreated cells, implying the direct role of PPRV-coded N protein in the induction of autophagy. Additionally, lysotracker dyes can also be employed as markers to visualize lysosomes and the autophagic flux. The number of red-colored puncta were found to be increasing with the increasing expression of PPRV-N protein in the presence of chloroquine, which suggests that the effect of PPRV-N on punctate formation is dose-dependent. The cells with larger number of red puncta are indicative of increased autophagic activity, thereby further confirming the role of PPRV-N in the induction of the autophagy process.

**Figure 8:**
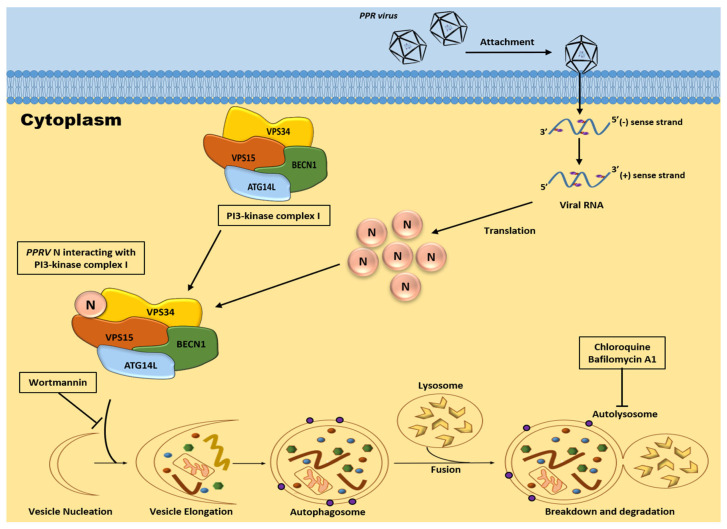
Schematic representation of how PPRV−N protein triggers autophagy via interaction with phosphatidylinositol−3−kinase complex−I. The inhibition of PI3−kinase complex with wortmannin stops autophagy. *Peste des petits ruminants*-virus-coded structural N protein interacts with the components of phosphatidylinositol−3−kinase complex−I and is associated with the VPS34 protein of PI3K complex−I. The physical stable interaction between PPRV−N and PI3K complex−I is coincident to the initiation of the process of autophagy, resulting in the formation of autophagosomes, which later fuses with lysosomes to form autolysosomes. Wortmannin inhibits phosphatidylinositol−3−kinase complex−I by blocking the production of PI3P, which is essential for the initiation of autophagy via the recruitment of other ATG proteins at the isolation membrane or phagophore. The disruption of the interaction between PPRV−N and PI3K complex−I proteins aborts the process of autophagy initiation. Our model proposes that PPRV−N induces autophagy in cells, which is mediated mainly by phosphatidylinositol−3−kinase complex−I.

Autophagosomes are defined as double-membrane structures and can be visualized at the ultrastructural level using transmission electron microscopy (TEM). A study had employed TEM to show that Dengue Virus-2 can induce autophagosome formation, as shown by ultra-structure images [54]. Some studies have shown that PPR virus infection induces two distinct waves of autophagy, which have been confirmed via the ultrastructural analysis of EECs infected with UV-irradiated PPRV or PPRV using TEM [28]. Our study investigates the presence of autophagosomes in PPRV-N-expressing HEK-293T cells and also in rapamycin-treated cells. Many double-membrane autophagosomes and single-membrane autolysosomes were found within these PPRV-N-expressing and rapamycin-treated cells. Completely or partially degraded organelles were seen in the cells with active autophagy. Therefore, this confirms that PPRV-N promotes autophagy in cultured cells. Our study therefore provides evidence for the critical role of PPRV-N protein in the induction of autophagy, which is likely to be mediated by phosphatidylinositol-3-kinase complex-I (Figure 8).

## Data Availability

Data are contained within the article or Supplementary Material.

## Supplementary Materials

The following supporting information can be downloaded at: https://www.mdpi.com/article/10.3390/v15091805/s1. Table S1: The list of primer pairs for the cloning of different genes used in this study; Table S2: The list of qPCR primer pairs for different target genes used in this study.
