# Supplementary material for: Nucleocapsid Protein (N) of Peste des petits ruminants Virus (PPRV) Interacts with Cellular Phosphatidylinositol-3-Kinase (PI3K) Complex-I and Induces Autophagy
Source: Viruses. 2023 Aug 24;15(9):1805. doi: 10.3390/v15091805 (PMC10536322; doi:10.3390/v15091805)
Supplement: Supplementary file 1 [file viruses-15-01805-s001.zip › viruses-2545917-supplementary.pdf]

viruses

MDPI

Article

Nucleocapsid Protein (N) of *Peste des petits ruminants* Virus (PPRV) Interacts with Cellular Phosphatidylinositol-3-Kinase (PI3K) Complex-I and Induces Autophagy

Yash Chaudhary[1], Juhi Jain[1], Sharad Kumar Gaur[1], Prabhakar Tembhurne[2], Shanmugam ChandraSekar[3], Muthuchelvan Dhanavelu[3], Sharvan Sehrawat[4] and Rajeev Kaul[1] *

[1] Department of Microbiology, University of Delhi South Campus, New Delhi, India; yash-chaudhary0210@gmail.com; jjain3101@gmail.com; sharadgaur992@gmail.com
[2] Department of Microbiology, Nagpur Veterinary College, Nagpur, India; prabhakar.tembhurne@gmail.com; schand_vet@yahoo.co.in
[3] Division of Virology, Indian Veterinary Research Institute, Mukteshwar, India; drchelva@gmail.com
[4] Indian Institute of Science Education and Research, Mohali, India; sharvan@iisermohali.ac.in
* Correspondence: rkaul@south.du.ac.in; Tel.: 011-24157368

Abstract: Autophagy is an essential and highly conserved catabolic process in cells which is important in the battle against intracellular pathogens. Viruses have evolved several ways to alter the host defense mechanism. PPRV infection is known to modulate the components of host cell defense system resulting in enhanced autophagy. In this study, we demonstrate that N protein of PPRV interacts with the core components of the class III phosphatidylinositol-3-kinase (PI3K) complex-I and results in induction of autophagy in the host cell over-expressing this viral protein. Our data shows the interaction between PPRV-N protein and different core components of autophagy pathway i.e. VPS34, VPS15, BECN1 and ATG14L. The PPRV-N protein can specifically interact with VPS34 of the PI3K complex-I, and co-localize with the proteins of PI3K complex in the same subcellular compartment, in the cytoplasm. These interactions did not affect the intracellular lo- calization of the different host proteins. The autophagy-related genes were transcriptionally mod- ulated in PPRV-N expressing cells. The expression of LC3B and SQSTM1/p62 was also modulated in PPRV-N expressing cells indicating the induction of autophagic activity. The formation of typi- cal autophagosomes with double membranes was visualized by transmission electron microscopy in PPRV-N expressing cells. Taken together, our findings provide the evidence for a critical role of N protein of PPR virus in the induction of autophagy likely to be mediated by PI3K complex-I of the host.

Keywords: *Peste des petits ruminants* virus (PPRV); PPRV-N; PI3K complex-I; Autophagy; BECN1

Supplementary Data:

Table S1. The list of primer pair for cloning of different genes used in the study.

Target gene	Primers (5'-3')	Restriction sites
PPRV N-Flag	Fwd: TTGAATTCCACCATGATTAAAAGCTTAGCATTG	EcoRI
	Rev: TTTGCGGCCGCATCCTTGTCGTTGTAG	NotI
GST-PPRV-N	Fwd: TTGATATCAATGGCGACTCTTATTAAAA	EcoRI
	Rev: TTGCGGCCGCTTATCCTTGTCGTTGTAGACC	NotI

HA-BECN 1	Fwd: TTAAGCTTCCACCATATGGAAGGGTCTAAGA<br>Rev: TTGAATTCGGCTTTAGCTAGCTCATTTG	EcoRI<br>HindIII
GST-BECN 1	Fwd: TTGGATCCACCATGGAAGGTCTAAGACG<br>Rev: TTGTCGACGGCTTTAGCTAGCTCATTTGTTAT	BamHI<br>SalI
HIS-BECN 1	Fwd: TTCATATGATGGAAGGGTCTAAGACG<br>Rev: TTGGATCCTCATTTGTTATAAAATTG	NdeI<br>BamHI
HA-ATG14L	Fwd: TTTGAATTCCACCAATGGCGTCTCCCAGT<br>Rev: TTTCTAGATTAACGGTGTCCAGTGTAAG	EcoRI<br>XbaI

Table S2. The list of qPCR primer pair for different target genes used in the study.

**Target gene**	**Primers (5'-3')**
TFEB	Fwd: ATCACCTGGACTTCAGCCAC<br>Rev: AGTGAGTCGTCCAGGAGCAT
E2F1	Fwd: ATGAAGCTAAGAAAGCTGCA<br>Rev: TTCTTCTTTAGGTTGAT
BECN1	Fwd: GGACACTCAGCTCAACGTCA<br>Rev: TTTCCACGTCTTCCAGCTCC
FOXO	Fwd: GCTGCATCCATGGACAACAACA<br>Rev: CGAGGGCGAAATGTACTCCAGTT
GATA 4	Fwd: ATGCCTTTACACGCTGATGG<br>Rev: ATGCAAAACCCACGGTCTAG
ZKSCAN	Fwd: CAGTGCAGTATGTGTGGCAA<br>Rev: TTTTCCAACTGGCTTTCCAT
GAPDH	Fwd: AAGGTGAAGGTCGGAGTCAACG<br>Rev: CCTTCTCCATGGTGGTGAAGAC
